# Disparities in transplant access and outcomes after first cirrhosis decompensation in alcohol-related liver disease

**DOI:** 10.1016/j.jhepr.2025.101594

**Published:** 2025-09-18

**Authors:** José Ursic Bedoya, Charlotte De Choudens, Margaux Delhomme, Astrid Herrero, Stéphanie Faure, Lucy Meunier, Magdalena Meszaros, Georges-Philippe Pageaux, Claire Duflos

**Affiliations:** 1 Department of Hepatogastroenterology, Hepatology and Liver Transplantation Unit, Saint Eloi Hospital, Institut de Génétique Moléculaire de Montpellier, University of Montpellier, CNRS, Montpellier, France; 2 Clinical Research and Epidemiology Unit, Department of Public Health, CHU Montpellier, University of Montpellier, France; 3 Department of Hepatogastroenterology, Hepatology and Liver Transplantation Unit, Saint Eloi Hospital, Montpellier, France; 4 Department of Hepato-Biliary and Pancreatic Surgery and Liver Transplantation, Montpellier University Hospital, Montpellier, France

**Keywords:** Liver transplantation, Cirrhosis, Healthcare disparities, Social determinants of health

## Abstract

**Background & Aims:**

Several studies have shown that non-medical factors can determine access to liver transplantation (LT). Most of these studies are from North America, where the healthcare system has specific features that may not be generalisable to Europe. We investigated the factors associated with access to LT in patients admitted for a first episode of decompensated cirrhosis in France and to determine potential treatment gaps according to disease aetiology.

**Methods:**

We conducted a retrospective cohort study using the French national health data system to identify patients hospitalised for a first decompensation of cirrhosis between 2015 and 2022, followed until December 2023. We built a frailty model to search for factors associated with access to LT and to quantify inter-centre variability. Then, we compared survival rates and incidence of liver-related death according to disease aetiology, including competing risk analyses.

**Results:**

We identified 65,771 patients (73.5% men, 82.3% alcohol-related liver disease [ALD]), with a mean follow-up time of 36 months. Of these patients, 3,596 (5.5%) were transplanted and 35,660 (54.2%) died. Patients with ALD were less likely to be transplanted (hazard ratio [HR] 0.75, 95% CI 0.69–0.81, *p* <0.0001), as were women (HR 0.75, 95% CI 0.69–0.81, *p* <0.0001), patients with solidarity-based health insurance (HR 0.82, 95% CI 0.74–0.90, *p* <0.0001) and those from socially deprived backgrounds (HR 0.97, 95% CI 0.94–0.99, *p* = 0.01). Restricted LT access for patients with ALD was associated with lower survival rates and increased liver-related mortality compared with non-ALD patients.

**Conclusions:**

Our study highlights several factors contributing to unequal access to LT in France. Reduced access for patients with ALD suggests the presence of structural stigma.

**Impact and implications:**

This study provides the first national-level evidence from a European country showing that patients with alcohol-related cirrhosis, particularly women and those from socioeconomically disadvantaged backgrounds, experience lower access to liver transplantation and higher liver-related mortality. These findings could be important for clinicians, researchers, and health policymakers seeking to improve equity in transplant access within universal healthcare systems. While our study does not capture all clinical or psychosocial determinants of transplant eligibility, it highlights systemic disparities that could be mitigated through national referral protocols, standardised patient evaluations, and targeted support for vulnerable populations. Future work should integrate clinical data and patient-reported outcomes to further refine these observations and guide practice change.

## Highlights

• Nationwide cohort study on liver transplant access after decompensation of cirrhosis.
• Alcohol-related cirrhosis is linked to lower transplant rates and higher mortality.
• Female sex and social deprivation independently reduce transplant probability.
• Structural barriers, not centre bias, may explain inequities in transplant access.

## Graphical abstract

**Figure:**
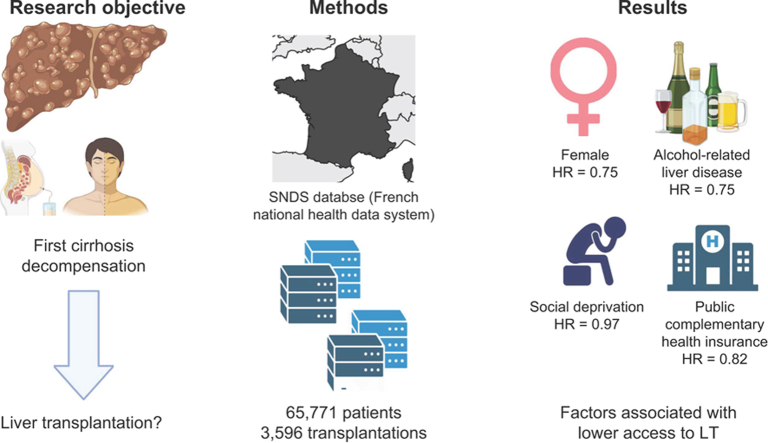

## Introduction

Liver transplantation (LT) is the only curative treatment for end-stage liver disease. This intervention offers excellent long-term results, even for the most severe cases.^1^^,^^2^ In a context of organ shortage, where only a minority of patients in need will receive a graft,^3^ equity is one of the cornerstones of LT. Referral to a transplant centre should only be based on medical conditions, but some barriers to referral have been identified in the USA and Canada. For example, the most socially disadvantaged patients are less likely to be on the waiting list, and less likely to receive transplants.[3], [4], [5] Belonging to an ethnic minority is also a disadvantage: several studies have shown a decreased access to transplantation in African-American and Hispanic patients compared with White patients.^3^^,^^6^^,^^7^ A greater distance between home and the nearest transplant centre is also a loss of opportunity for wait listing and transplantation.^8^ Finally, an American study including 34,494 patients with cirrhosis between 2011 and 2017 demonstrated that alcohol-related liver disease (ALD) aetiology, despite good post-transplant survival,^1^ is a barrier at every stage: referral to the transplant centre, wait listing and transplantation.^3^ However, it is unclear whether this referral bias is homogeneous between centres. Recently, a study examined data on every patient with ALD assessed for LT in the United Kingdom during a 12-month period found that women and patients from the most deprived deciles were relatively underrepresented in the transplant list.^9^

In this context, data have mainly come from North American centres, where the healthcare system has certain specific features that are difficult to generalise to Europe. In France, the entire population has access to a universal, tax-funded healthcare system (*Assurance Maladie*). Moreover, social dynamics are not identical between the two regions and the epidemiology of cirrhosis also differs with, for example, more metabolic dysfunction-associated steatohepatitis (MASH) in the USA than in France,^3^ where alcohol-related cirrhosis is more prevalent.^10^

To the best of our knowledge, no study has yet provided information on the factors contributing to inequalities in access to LT in Europe. The aim of our study is to investigate the factors associated with access to LT in patients admitted to hospital for a first episode of decompensated cirrhosis and to determine whether there are treatment gaps due to the aetiology of the underlying liver disease.

## Materials and methods

This study is an analytical retrospective cohort based on the French national health data system (*Système National des Données de Santé,* SNDS), which gathers several databases described below. Using the SNDS provides access to all hospital stays; since patients with decompensated cirrhosis are almost systematically hospitalised, we used this data to identify incident cases between 2015 and 2022 exhaustively and study their care pathway until transplantation. Our study was approved by our institutional review board (IRB Adène, Number IRB_ADENE_20240102). This study is based on fully anonymized data from the SNDS, which includes routinely collected administrative and medical records. According to French regulations, studies using SNDS data do not require individual patient consent, as the data are de-identified and used for public health research purposes. Patients and the public were not involved in this research.

### Data sources

We used data from the French National Hospital Discharge Database (*Programme de Médicalisation des Systèmes d’Information*, PMSI), which covers all hospitals stays in France.^11^ Each stay is coded with a principal diagnosis, to which may be added a related diagnosis and one or more associated diagnoses. These diagnoses are coded according to ICD-10. Medical procedures and acts performed at the hospital are also available, coded according to the French Classification of Medical Procedures (CCAM).

Pathology and expenditure mapping^12^ are data supplied by the *Assurance maladie* coded using data from the SNDS. It describes patients' comorbidities based on the diagnostic codes of hospital stays, long-term conditions declared by physicians to the *Assurance Maladie*, and the dispensing of certain drugs. It also contains an ecological deprivation index, the FDEP15 (update of FDEP09).^13^ We used the latest available version (G10) of pathology and expenditure mapping.

The Inter-regime Consumption DataMart (DCIR) contains health insurance reimbursement data for ambulatory care as outpatient consultations, treatment supply (coded according to the Anatomical Therapeutic Chemical [ATC] code), long-term illness and general practitioner (GP) declarations by patients (*i.e.* adherence to the gate-keeping role of GPs, which allows fees reductions).

Finally, the Centre for Epidemiology of Medical Causes of Death provides data on dates and causes of death in France until December 31, 2022. We used the DCIR data to extract subsequent dates of death. Linking method between databases is detailed in Fig. S1.

### Study cohort

We included patients aged between 18 and 70, living in France, hospitalised for a first decompensation of cirrhosis between January 1^st^, 2015 and December 31^st^, 2021. We reported their outcomes up to December 31^st^, 2023. The date of inclusion was the first day of hospitalisation. We selected only patients under 70 years old, as this threshold is the age limit usually used by teams for access to LT. Patients who had been previously hospitalised for decompensated cirrhosis or who had undergone LT between 2010 and their inclusion date (to have at least 5 years of hindsight) were excluded.

We defined hospitalisation for decompensated cirrhosis as follows: main diagnosis corresponding to a diagnosis of fibrosis, cirrhosis or cause of cirrhosis and at least one associated or related diagnosis of an event defining cirrhosis as decompensated; or main diagnosis corresponding to an event defining cirrhosis as decompensated and at least one associated or related diagnosis of fibrosis, cirrhosis or cause of cirrhosis.

Events defining cirrhosis as decompensated were: ascites, hepatorenal syndrome, sepsis, bleeding from oesophageal or gastric varices, non-occlusive jaundice and hepatic encephalopathy^14^ (see Table 1 for detailed codes). Outpatient data (*i.e.* outpatient paracentesis, HCC diagnosis without acute decompensation needing hospitalisation) were not considered. Sepsis codes were included following Baveno VII Consensus Conference statements 5.10 to 5.12^15^ and previous literature showing that adding sepsis codes adds exhaustivity in identifying ALD admissions through ICD-10 coding.^16^^,^^17^ Details on the events defining cirrhosis as decompensated can be found on Table S2.

**Table 1:** ICD-10 codes used.

ICD-10 codes	Disease
K700–K704, K709–K719, K721, K729–K746, K758–K760, K768, K769, K778, B180–B182, B188, B189, E831	Diagnosis of fibrosis, cirrhosis or cause of cirrhosis
A418, A419, E807, G934, G92, G93, G94, G96, G98, G99, I850, I983, K650, K658, K659, K767, K922, R170, R18, R258, R400-R402, R410, R418	Event defining cirrhosis as decompensated
F100, K852, Y910, Y911, Y919	Acute alcohol-related condition
K700–K704, K709, E512, F101–F109, G312, G621, G721, I426, K292, K860, T510, T518, T519, Y912, Y913, Z714, Z502	Chronic alcohol-related condition
B180, B181	Hepatitis B virus
B182	Chronic viral C hepatitis
K758	MASH

### Primary outcome

The primary endpoint was LT. Patients were considered transplanted if they were admitted to the hospital with one of the codes corresponding to LT (CCAM codes HLEA001, HLEA002, HGEA002 and HGEA004), between their inclusion date and December 31^st^, 2023. The transplant date was defined as the day of admission.

### Covariates and confounders

France provides universal, tax-funded healthcare coverage to all legal residents through a national health insurance system. However, copayments are usually required at the point of care. These are typically covered either by private complementary insurance or, for low-income individuals, by a public complementary scheme (‘Complémentaire santé solidaire’, named “Beneficiary of public complementary health insurance”), which ensures full coverage without out-of-pocket expenses.

The main aetiologies of cirrhosis in France are ALD, HBV, HCV and MASH.^18^ They are not mutually exclusive. In the absence of any of these four conditions, the patient was considered to have another, unspecified, aetiology of cirrhosis. Alcohol-related cirrhosis was defined as at least two hospitalisations for an acute alcohol-related condition or at least one hospitalisation for a chronic alcohol-related condition in the 5 years preceding the first hospital stay for decompensated cirrhosis, including the index stay.

HBV or MASH cirrhosis were defined by at least one hospital stay with a principal, related or associated diagnosis linked respectively to chronic hepatitis B or MASH since 2010 and up to 1 year after their first hospitalisation for decompensated cirrhosis. We included stays after the index stay, as a diagnosis of HBV or MASH may be made as a result of the aetiological investigation carried out during this stay. For HCV, we used the variable corresponding to the presence of active or cured hepatitis C from the pathology and expenditure mapping for the year of the patient's initial hospitalisation. All ICD-10 codes used are shown in Table 1.

We calculated an age-adjusted Charlson comorbidity index as calibrated by Bannay *et al.*^19^ with comorbidity extracted from pathology and expenditure mapping and from the PMSI data. The algorithm used to construct the index is detailed in Table S1.

The FDEP15 is a variable calculated at the smallest administrative level, based on the proportion of working-class individuals, the unemployment rate, the proportion of high school graduates in the population aged over 15 years, and household income. A higher FDEP15 means a more deprived status.^13^

In the SNDS database, socio-demographic information includes a variable indicating the sex of individuals. This variable is generally coded in binary form to distinguish between men and women. It is important to note that the SNDS records gender as declared in administrative or medical documents, without explicitly distinguishing between biological gender and perceived or social gender. Consequently, the available data reflects the administrative sex of individuals, as recorded in the health and health insurance systems.

We classified every index hospital stay structure in four categories: centres performing LT, university hospitals (excluding transplant centres), other public hospitals and private hospitals.

We used multiple items to assess the quality of medical follow-up. First, we identified whether the patient declared an attending physician at the time of index hospital stay. In France, it is not compulsory to declare an attending physician. However, it does enable better reimbursement of medical care, particularly consultations with specialists. We also identified the declaration of long-term illness for cirrhosis in progress at the time of hospitalisation. This declaration is not mandatory in France either, but it does entitle the bearer to a financial waiver for treatment related to the associated pathology. In addition, we extracted medical consultations with a GP and with a gastroenterologist in the 12 months prior to admission to hospital. We also evaluated the follow-up of alcohol addiction. We identified hospital stay for alcohol rehabilitation from the PMSI’s tables using the ICD-10 code Z502. We selected stays in the 12 months before or after the first hospitalisation for decompensated cirrhosis. We also extracted data on drug treatment for alcohol dependence in the 12 months before and after the hospitalisation. We selected two types of ATC codes: all the codes beginning with “N07BB” which is used for the category “drugs used for alcohol dependence” (including disulfiram, calcium carbimide, acamprosate, naltrexone and nalmefene) and the code M03BX01 corresponding to baclofen, a muscle relaxant widely used in alcohol dependence.

### Statistical analyses

We described our complete baseline population according to transplant status. Quantitative variables were described by their median, quartiles, mean and standard deviation. Categorical variables were described by their number and percentage. We also described the hospital centres where patients were included.

We used a frailty model to analyse the variables affecting access to LT. A frailty model is an extension of the Cox proportional hazards model that accounts for unobserved heterogeneity and clustering effects. In this study, we used a shared frailty model with a random effect at the transplant centre level to quantify inter-centre variability in liver transplantation access. This approach allows us to adjust for measured confounders while capturing unexplained centre-specific differences in transplantation likelihood. Applying a random effect from the gamma distribution to the centre allowed us to use the frailty variance as a reflection of the inter-centre variability not explained by our model. We created two models: one with the alcohol-related cirrhosis variable and one without. We then compared their frailty variance, which allowed us to quantify the inter-centre variability explained by alcohol-related cirrhosis.^20^

The event was LT. Patients were censored on December 31st, 2023, or at their date of death. We tested the proportional hazards assumption for each variable and the linearity hypothesis for continuous variables. We preselected the variables that had a *p* value <0.10 in the univariate models. Variables were then selected using the backward method.

We used the Kaplan-Meier method to estimate survival probabilities from inclusion date until death and their pointwise 95% CIs. The Log-rank non-parametric test for comparison of survival distributions was used to compare survival differences between groups. The alpha risk was set to 5.0%. We also performed a competing risks analysis to estimate the cumulative incidence of liver-related death, considering death from other causes as a competing event. Patients were classified into four groups based on liver disease aetiology (alcohol-related *vs*. non–alcohol-related cirrhosis) and liver transplantation status (transplanted *vs.* non-transplanted). Cumulative incidence functions were compared across groups using Gray’s test.

To explore whether certain liver transplant centres had significantly higher or lower rates of transplantation for alcohol-related cirrhosis than expected, we conducted an indirect standardisation analysis. For each centre, we calculated the expected number of liver transplants based on the clinical and demographic characteristics of its patients, using the variables that were independently associated with transplantation in our frailty model. We then compared the observed *vs.* expected transplant incidence across centres and plotted the difference (residual variation) by deciles of expected incidence. This approach allowed us to visualize unexplained inter-centre variability, potentially reflecting differences in local practices or attitudes toward transplantation in ALD. The analysis was restricted to patients with alcohol-related cirrhosis. Statistical analyses were performed using SAS 8.3 and R 4.3.1.

## Results

### Study population

We included 65,771 patients corresponding to our inclusion criteria, with a mean follow-up time of 36 months and 195,270 person-years. Of these patients, 73.48% were men and 82.26% had alcohol-related cirrhosis. By December 31st, 2023, 3,596 (5.47%) had been transplanted and 35,660 (54.22%) had died. Transplant patients had a median time to transplant of 319 days. Of these, only 152 LT (4.2%) occurred during the index hospitalisation.

Transplant patients were on average younger (54.83 *vs.* 57.5 years, *p* <0.0001) and had a trend to a lower mean Charlson score (3.93 *vs.* 4.34, *p* <0.0001) than non-transplant patients. They were also less socioeconomically disadvantaged (Fdep15 at 0.12 *vs.* 0.42, *p* <0.0001 and public complementary health insurance in 16.53% *vs.* 18.07%, *p* = 0.02) and lived closer to a transplant centre (80.65 km *vs.* 90.34 km, *p* <0.0001). Overall, they had better medical follow-up, with 48.08% having a long-term illness declaration at the time of hospitalisation (*vs.* 27.64% for non-transplant patients, *p* <0.0001) and 42.74% having had at least one consultation with a gastroenterologist during the year (*vs*. 30.18%, *p* <0.0001). On the other hand, there was no significant difference in the declaration of the attending physician (67.96% *vs.* 72.86%, *p* <0.0001, Table 2).

**Table 2:** Patient characteristics at baseline.

	Overall population N = 65,771	Non-transplant patients n = 62,175	Transplant patients n = 3,596	*p* values
**Socio-demographic characteristics**				
Men	48,329 (73.48)	45,620 (73.37)	2,709 (75.33)	**0.01**
Age	57.36 ± 9.33 59.00 [52.00–65.00]	57.51 ± 9.29 59.00 [52.00–65.00]	54.83 ± 9.62 57.00 [50.00–62.00]	**<0.0001**
Deprivation index FDEP15 (n = 61,089)	0.41 ± 1.55 0.50 [-0.49 to 1.45]	0.42 ± 1.55 0.51 [-0.46 to 1.46]	0.12 ±1.59 0.23 [-0.81 to 1.22]	**<0.0001**
Beneficiary of public complementary health insurance (n = 62,431)	11,226 (17.98)	10,660 (18.07)	566 (16.53)	**0.02**
Distance from home to nearest transplant centre (km) (n = 63,118)	89.80 ± 64.51	90.34 ± 64.45	80.65 ± 64.96	**<0.0001**
	83.52 [31.08–137.12]	84.63 [31.86–137.57]	68.67 [21.36–127.55]	

**Clinical characteristics**				
Charlson index (n = 63,497)	4.31 ±2.25 3.00 [3.00–5.00]	4.34 ± 2.29 3.00 [3.00–5.00]	3.93 ±1.23 3.00 [3.00–5.00]	**<0.0001**
Alcohol-related cirrhosis	54,110 (82.26)	51,430 (82.72)	2,680 (74.53)	**<0.0001**
Hepatitis C virus (n = 63,497)	4,252 (6.70)	3,894 (6.49)	358 (10.33)	**<0.0001**
MASH	837 (1.27)	751 (1.21)	86 (2.39)	**<0.0001**
Hepatitis B virus	978 (1.49)	901 (1.45)	77 (2.14)	**<0.0001**
Other aetiology	8,662 (13.17)	8,022 (12.90)	640 (17.80)	**<0.0001**
Deceased	35,660 (54.22)	35,010 (56.31)	650 (18.08)	**<0.0001**
Medical care at the time of index hospital stay				
Declaration of long-term illness	18,917 (28.76)	17,188 (27.64)	1,729 (48.08)	**<0.0001**
Attending physician declaration	47,742 (72 0.59)	45,298 (72.86)	2,444 (67.96)	**<0.0001**
At least one gastroenterologist consultation in the previous year	20,302 (30.87)	18,765 (30.18)	1,537 (42.74)	**<0.0001**
At least three consultations with a general practitioner in the previous year	46,095 (70.08)	43,579 (70.09)	2,516 (69.97)	0.87
At least one stay for alcohol withdrawal in the previous year	2,211 (3.36)	2,121 (3.41)	90 (2.50)	**0.003**
At least one delivery of treatment for alcohol dependence in the previous year	6,193 (9.42)	5,941 (9.56)	252 (7.01)	**<0.0001**
Type of initial hospitalisation centre				**<0.0001**
Liver transplant centre	8,954 (13.61)	7,950 (12.79)	1,004 (27.92)	
University hospital	10,794 (16.41)	10,117 (16.27)	677 (18.83)	
Other public hospital	37,326 (56.75)	35,820 (57.61)	1,506 (41.88)	
Private hospital	8,697 (13.22)	8,288 (13.33)	409 (11.37)	
Medical care after first hospitalisation				
At least one stay for alcohol withdrawal in the following year	2,410 (3.66)	2,331 (3.75)	79 (2.20)	**<0.0001**
At least one delivery of treatment for alcohol dependence in the following year	4,381 (6.66)	4,223 (6.79)	158 (4.39)	**<0.0001**

Data are expressed as number (%) or as mean ± standard deviation and median [Q1-Q3]. *p* values are the results of chi^2^ test for qualitative variables or *t* test for quantitative variables. *p* < 0.05 is significant (bolded values). FDEP, French Deprivation Index; MASH, metabolic dysfunction-associated steatohepatitis.

At the time of their first hospitalisation for decompensated cirrhosis, patients were admitted to a total of 1,031 centres. Over 7 years, each centre managed between 1 and 844 included patients, with a median of 19 patients. There were 18 transplant centres (1.75%) that cared for 13.61% of patients and 44 university hospital centres (4.27%) that admitted 16.41% of our patients (Table 3). The lower section of Table 3 presents the mean characteristics of patients at the centre level. Each value reflects the average across centres, summarizing the typical profile of patients admitted per centre.

**Table 3:** Centre characteristics at baseline.

Characteristics	
Type of centre	
Public hospital	486 (47.14)
Private hospital	483 (46.85)
University hospital	44 (4.27)
Liver transplant centre	18 (1.75)
Median number of patients	19.00 [4.00–70.00]
Number of patients	
1 to 5	305 (29.58)
6 to 30	304 (29.49)
31 to 100	225 (21.82)
101 to 400	165 (16.00)
401 to 844	32 (3.10)

**Mean characteristics of patients at the centre level**	
Mean age	58.49 ± 4.54
Percentage of men	71.96 ± 22.59
Mean FDEP15	0.16 [−0.59 to 0.74]
Mean Charlson index	4.51 ± 1.62
Percentage of patient with alcohol-related cirrhosis	85.16 [71.87–93.69]
Percentage of patients with hepatitis C virus	2.08 [0.00–7.14]
Percentage of patients with MASH	0.00 [0.00–0.40]
Percentage of patients with hepatitis B virus	0.00 [0.00–0.54]
Percentage of patients with none of the above aetiologies	10.84 [3.39–21.26]
Percentage of patients with declaration of long-term illness	27.27 [16.67–33.74]
Percentage of patients with at least one gastroenterologist consultation in the previous year	28.57 [17.09–57.89]
Percentage of patients transplanted	1.81 [0.00–5.51]

Data are expressed as number (%) or as mean ± standard deviation or median [q1-q3]. FDEP, French Deprivation Index; MASH, metabolic dysfunction-associated steatohepatitis.

### Frailty model

We included 60,071 of the 65,771 patients in our model because of missing data in the pathology and expenditure mapping, for a total of 3,326 LT.

As shown in Table 4, higher age, Charlson index and FDEP15, female sex, having an alcohol-related cirrhosis and being a beneficiary of public complementary health insurance were statistically associated with less access to transplantation in the multi-adjusted model. The continuous deprivation index was statistically associated with LT (HR 0.97; 95% CI 0.94–0.99, *p* = 0.01).

**Table 4:** Frailty model: Association between covariates and liver transplantation, N = 60,071.

	Univariate model			Multivariate model		
	Hazard ratio	95% CI	*p* values	Hazard ratio	95% CI	*p* values
Age	0.98	0.98–0.99	**<0.0001**	0.98	0.98–0.99	**<0.0001**
Female sex	0.84	0.78–0.91	**<0.0001**	0.75	0.69–0.81	**<0.0001**
Fdep15	0.91	0.89–0.92	**<0.0001**	0.97	0.94–0.99	**0.01**
Beneficiary of public complementary health insurance	0.81	0.74–0.88	**<0.0001**	0.81	0.74-0.90	**<0.0001**
Distance from home to nearest transplant centre (10 km)	0.98	0.98–0.99	**<0.0001**			
Charlson index	0.98	0.96–1.00	**0.09**	0.98	0.96–1.00	**0.047**
Alcohol-related cirrhosis	0.65	0.60–0.70	**<0.0001**	0.75	0.69–0.81	**<0.0001**
Hepatitis C virus	1.66	1.49–1.85	**<0.0001**			
MASH	2.01	1.62–2.49	**<0.0001**	1.46	1.17–1.83	**0.001**
Hepatitis B virus	1.50	1.19–1.88	**0.0005**			
Declaration of long-term illness	2.37	2.22–2.53	**<0.0001**	2.17	2.03–2.33	**<0.0001**
Declaration of attending physician	1.05	0.98–1.13	0.17			
At least three consultations with a general practitioner in the previous year	1.18	1.09–1.26	**<0.0001**			
At least one gastroenterology consultation in the previous year	1.94	1.82–2.08	**<0.0001**	1.71	1.59–1.83	**<0.0001**

**Type of centre (reference = general public hospital)**	**<0.0001**			**<0.0001**		
Liver transplant centre	2.85	2.63–3.09	**<0.0001**	2.26	1.91–2.67	**<0.0001**
University hospital without LT program	1.55	1.41–1.70	**<0.0001**	1.48	1.28–1.71	**<0.0001**
Private hospital	1.16	1.04–1.29	**0.008**	0.93	0.82–1.05	0.25

Bolded *p* values are significant (*p* <0.05) in the frailty statistical model. MASH, metabolic dysfunction-associated steatohepatitis.

**Fig. 1:**
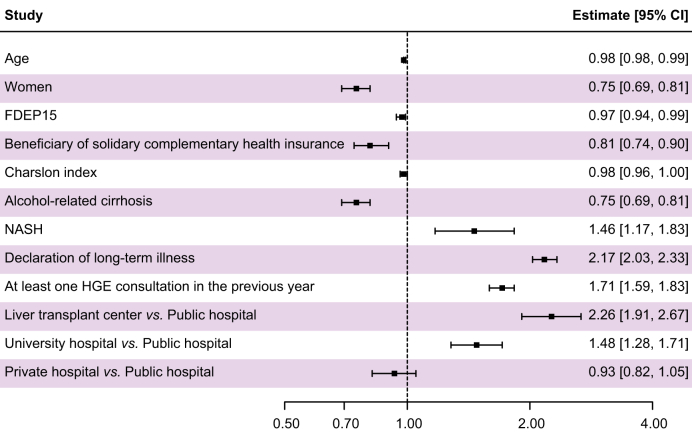
Forest Plot of the multi-adjusted frailty model showing association between covariates and liver transplantation. Representation of the hazard ratio with their 95% CIs. The x-axis is logarithmically scaled. HGE, hepato-gastroenterology; MASH, metabolic dysfunction-associated steatohepatitis.

On the other hand, a better follow-up before the first hospitalisation (*i.e*. a declaration of long-term illness or at least one gastroenterology consultation in the previous year) was associated with a better chance of receiving a transplant. An index hospital stay in a transplant centre or university hospital at the time of first decompensation, rather than another public hospital, was also associated with better chances of transplantation (Fig. 1).

The residual covariance of our model was 0.079. Removing alcohol-related cirrhosis, the covariance was 0.086, indicating that the inter-centre variability is only slightly explained by the alcohol-related background of the patients. This finding supports that poorer access to LT for patients with alcohol-related cirrhosis is likely to be a general problem and not related to centre-specific policies.

For details on model construction and sensitivity analyses, refer to the supplementary methods and Table S3. To ensure the robustness of MASH data, we also ran a frailty model defining MASH using the K758 code and presence of diabetes according to pathology and expenditure mapping (Table S4).

### Standardised incidences

To explore potential centre-level disparities in LT for alcohol-related cirrhosis, we analysed the 50% of centres with the highest ALD patient volume (484 centres, 47,435 patients). We compared observed *vs.* expected LT incidence for each centre, adjusting for patient characteristics using our frailty model.

We found substantial variation in observed LT incidence across centres, ranging from 0 to 105 transplants per 1,000 person-years (PY), while expected incidence ranged from 4.4 to 68.8 per 1,000 PY. The difference between observed and expected incidence varied widely, from –29.1 to +88 per 1,000 PY, suggesting important inter-centre disparities not explained by patient characteristics.

**Fig. 2:**
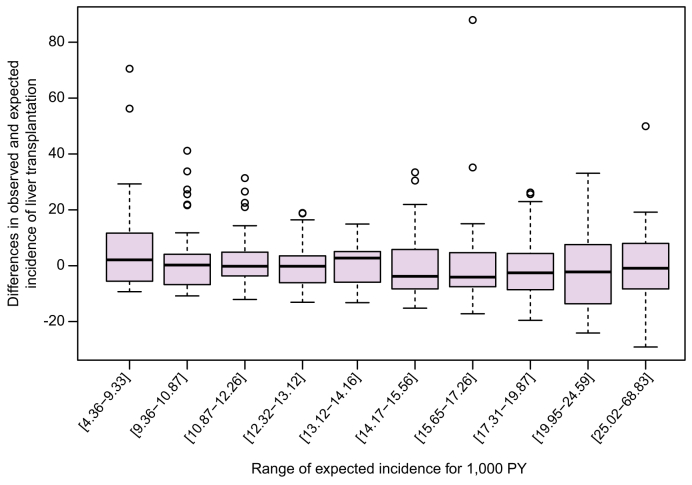
Differences in observed and expected incidences of liver transplant by decile of expected incidence for 1,000 PY. PY, person-year.

However, when we grouped centres by deciles of expected transplant incidence (*i.e.* patient case-mix, Fig. 2), we did not observe systematic patterns. In other words, centres treating patients with a higher likelihood of transplantation (based on clinical and demographic factors) did not consistently transplant more patients than others.

This suggests that inter-centre variability in LT access for ALD is limited, and that systemic or structural factors, such as uniform abstinence requirements or referral practices, may represent the main barriers, rather than centre-specific attitudes.

### Mortality and causes of death

**Fig. 3:**
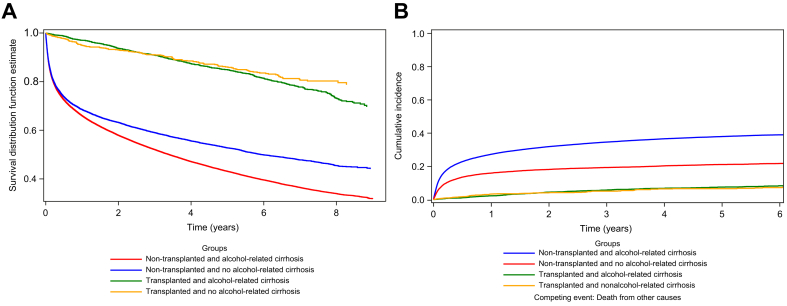
Comparison of survival and liver-related deaths according to transplantation status and cirrhosis aetiology. (A) Survival curves (log-rank test is significant *p* < 0.0001 between non-transplanted with alcohol-related cirrhosis *vs*. non-transplanted and no alcohol-related cirrhosis). (B) Cumulative incidence of liver-related death using a competing risks model (Gray test is significant *p* <0.0001).

The subsequent analysis investigated the potential correlation between reduced LT rates in patients with ALD and a possible phenomenon of recompensation following alcohol cessation. This hypothesis suggests that reduced mortality and liver-related mortality after the initial episode of decompensation could be indicative of recompensation. As shown in Fig. 3A, non-transplanted patients with alcohol-related cirrhosis had significantly lower survival than non-transplanted patients with cirrhosis of other aetiologies (log-rank test <0.0001). Most deaths were liver-related (excluding hepatobiliary cancer) in the non-transplanted/alcohol-related cirrhosis group (56.4% *vs*. 28.5% in the non-alcohol-related cirrhosis group, *p* <0.001), suggesting that a significant number of patients from the alcohol-related cirrhosis group could have benefited from LT (Table 5). To further explore liver-related mortality, we performed a competing risks analysis, considering non–liver-related deaths as competing events. As shown in Fig. 3B and Table S5, the cumulative incidence of liver-related death was highest in non-transplanted patients with alcohol-related cirrhosis, significantly exceeding that of all other subgroups (Gray’s test, *p* < 0.0001). These findings reinforce the observation that unmet transplant need in this population contributes substantially to early mortality.

**Table 5:** Causes of death.

Causes of death	Overall (N = 27,859)	Patient with non-alcohol-related cirrhosis (n = 4,064)	Patient with alcohol-related cirrhosis (n = 23,795)
Liver disease or complication related to cirrhosis, excluding cancer	14,577 (52.32%)	1,159 (28.52%)	13,418 (56.39%)
Malignant tumour of the liver and intrahepatic bile ducts	4,317 (15.50%)	821 (20.20%)	3,496 (14.69%)
Malignant solid tumours and haematological disease	2,920 (10.48%)	1,110 (27.31%)	1,810 (7.61%)
Cardiovascular disease	1,430 (5.13%)	223 (5.49%)	1,207 (5.07%)
Violent (including suicide) or accidental death	484 (1.74%)	48 (1.18%)	436 (1.83%)
Imprecise and unspecified causes of death	754 (2.71%)	118 (2.90%)	636 (2.67%)
Other causes	3,377 (12.12%)	585 (14.40%)	2,792 (11.73%)

∗*p* values of chi-squared tests are significant (<0.001) for every cause of death between the two groups tested here.

## Discussion

In this retrospective national cohort study, we found several characteristics associated with unequal access to LT among patients hospitalised for a first episode of decompensated cirrhosis. In particular, patients with alcohol-related cirrhosis were reported to have 25% less access to transplantation. This pattern seemed to be consistent across hospitals. We also confirmed that access to transplantation is determined by gender and social factors, even in a healthcare system based on solidarity and equity.

Our results are in line with the literature. The fact that older and more comorbid patients have less access to LT may be explained by a greater likelihood of having a contraindication to transplantation, but also by a reluctance on the part of physicians to transplant patients with a lower life expectancy. Patients with better pre-hospitalisation follow-up are more likely to receive transplants, indicating the importance of early and coordinated care.

Moreover, our data confirms that women have less access to LT than men, as previously reported in the literature. This disparity is multifactorial and likely stems from both biological and systemic factors. Biologically, women generally have lower muscle mass and creatinine levels compared to men, which can lead to underestimation of disease severity in scoring systems such as the traditional MELD score. This biological bias may result in lower prioritisation for transplantation despite comparable clinical needs.

Systemically, implicit biases in clinical decision-making and organ allocation processes could further disadvantage women. Addressing this issue requires a comprehensive approach, including the adoption of organ allocation policies that account for sex-based differences. Recent models such as MELD 3.0 or Gender-Equity Model for liver Allocation (GEMA),^21^^,^^22^ which incorporate adjustments for sex disparities, have shown promise in reducing these inequities.

In addition, it has been shown that women are less likely to discuss alcohol consumption with healthcare providers, which can delay interventions and potentially worsen ALD.[23], [24], [25] Beyond scoring modifications, awareness campaigns and education targeting healthcare providers are critical to mitigate implicit biases in the referral and evaluation process. Implementing these changes could lead to a more equitable liver transplantation system and improve outcomes for women suffering from advanced liver disease.

The lower access of the most deprived patients indicates that social determinants of health transcend healthcare system factors and cannot be explained solely by the healthcare system. Providing equal healthcare to the most disadvantaged patients is a major challenge, particularly in the setting of a life-saving procedure such as LT.

Our result showing a decreased access for patients with alcohol-related cirrhosis is also consistent with the literature. While it is plausible that LT was contraindicated in many patients in this group due to extrahepatic comorbidities (such as malignancy or mental health issues), the significantly higher liver-related mortality strongly suggests that some patients were not considered suitable for LT based on non-medical factors. Moreover, our frailty model is adjusted for Charlson, so we have evidence that for the same Charlson comorbidity index, patients with alcohol-related cirrhosis are less likely to be transplanted. The strength of the association between reduced access to LT and ALD is lower than the one found by Kanwal *et al.* in 2021.^3^ This can probably be explained in part by the higher rate of patients with an alcohol-related cirrhosis in France compared to the US, which could lead to lower stigma towards these patients.^26^ Prior data from the US, particularly the study by Giard *et al.*,^27^ showed that patients with ALD listed for transplantation had better wait-list outcomes compared to those with other aetiologies, including a lower risk of removal due to death or clinical deterioration and a higher likelihood of delisting for improvement. These results were interpreted as a consequence of the selection of ALD candidates with favourable prognoses, possibly related to mandated abstinence periods. In contrast, our population-based study highlights that a large proportion of patients with ALD are never transplanted and exhibit high liver-related mortality. One explanation could be that a proportion of patients is not listed, despite meeting the criteria for transplantation benefits, although this hypothesis cannot be verified since we lack the data on waitlisting. This discrepancy between wait-listed and general ALD populations emphasizes the need to re-evaluate current referral and listing practices, which may exclude high-risk patients who could benefit from timely transplantation.

Hence, our data suggests a potential structural stigma hindering access to LT in patients with ALD. During the study period, most transplant centres still used a policy including a certain duration of abstinence before listing, despite evidence showing the benefit of early access to LT in this setting. Furthermore, addiction management remains difficult to access for most patients.^28^ Yet, addiction treatment could enable some patients to enter the transplant evaluation process.

Our result showing higher LT probability for patients directly admitted to transplant centres seems logical: they are in direct contact with teams managing the procedures for accessing the waiting list and then transplantation. Access from non-transplant centres necessarily requires patients to be referred to a transplant centre, which can lengthen the time between admission and pre-transplant assessment and, ultimately, reduce patients' chances of accessing a graft in time. This is a very relevant finding given that transplant centres represented only a tiny proportion of the centres included in our study (1.8% of all centres).

Surprisingly, there does not seem to be a significant difference in access to LT for patients with ALD between centres. The distribution of incidence differences appears to be similar between centres with high and low expected transplant incidence. This finding highlights the fact that the structural stigma suffered by patients with ALD could be improved by national rather than centre-specific policy changes. For example, we can mention the implementation of a standardised evaluation of patients with ALD in need of transplantation regardless of the length of abstinence, as reported by Carrique *et al.*^29^ Additionally, establishing centralized multidisciplinary review boards to oversee transplant eligibility decisions could reduce local variability and implicit bias. Finally, systematically integrating addiction specialists into transplant evaluation teams could support individualized assessment of alcohol use disorder and recovery potential, thus facilitating equitable referral pathways.

To our knowledge, this is the first comprehensive large-scale study in France regarding determinants of access to LT. The use of a national cohort (SNDS) enabled us to create an exhaustive cohort without follow-up loss. However, there are a few limitations associated with the use of the SNDS. First, the lack of clinical data limits precise analysis and prevents us from fully adjusting our models to the health status of our patients. Our patient detection algorithm was built on a purely clinical basis and has not been validated for the detection of patients with decompensated cirrhosis, opening the door to a potential selection bias. In addition, as the initial aim of the PMSI databases is the public funding of hospitals, some secondary diagnoses may not be reported because they do not increase funding, or some coding may be imprecise due to similar funding between two or more similar codes.^11^

Furthermore, data from our registry lacked several key clinical variables such as MELD score, Child-Pugh class, or frailty indices – factors that are critical in assessing liver disease severity, prognosis, and transplant eligibility. This limitation is inherent to large-scale administrative datasets like the SNDS, which offer exceptional population coverage and longitudinal follow-up, but at the cost of reduced clinical granularity. While this trade-off limits precise adjustment for disease severity at the individual level, the broad scope of the cohort strengthens the external validity and generalisability of our findings. Another limitation of our study is the lack of access to the intermediate steps in the care pathway. Indeed, we had no access to the waiting lists of the French organ allocation agency (*Agence de la Biomédecine*). It would also have been useful to identify patients who had undergone a pre-transplant check-up, but we were unable to develop an efficient algorithm to detect these stays due to their heterogeneity (unpublished data from our University Hospital). Nevertheless, our primary endpoint, transplantation, is reliable. Indeed, this procedure is coded to classify the hospital stay and is associated with an important financial compensation. Therefore, it is systematically coded in the PMSI. The number of transplants per year that we found was consistent with data reported by the *Agence de la Biomédecine*.

In conclusion, this study highlights several factors that contribute to inequitable access to LT in France and underlines the need for a more equitable approach to the management of transplant candidates. The homogeneity of lower access for patients with ALD suggests the presence of structural stigma. Modification of national policies could lead to more equitable access to transplantation.

## Abbreviations

ALD, alcohol-related liver disease; ATC, Anatomical Therapeutic Chemical; CCAM, Classification Commune des Actes Médicaux (French Classification of Medical Procedures); DCIR, Inter-regime Consumption DataMart (French database for health insurance reimbursements); FDEP, French Deprivation Index; GP, general practitioner; HR, hazard ratio; LT, liver transplantation; MASH, metabolic dysfunction-associated steatohepatitis; PMSI, Programme de Médicalisation des Systèmes d’Information (French National Hospital Discharge Database); PY, person-year; SNDS, Système National des Données de Santé (French National Health Data System).

## Financial support

The authors did not receive any financial support to produce this manuscript.

## Authors’ contributions

Planning and conducting the study: JUB, CDC, CD. Interpreting data: all authors. Drafting the manuscript: CDC, JUB, GPP, CD.

## Data availability

The data supporting the findings of this study are derived from the French National Health Data System (SNDS). These administrative health data are not publicly available and were accessed under specific authorization. Access requests must be submitted to the Health Data Hub (formerly the National Institute for Health Data) and approved by the French Data Protection Authority (CNIL), in accordance with national regulations. Summary data may be available upon reasonable request to the corresponding author, subject to necessary permissions.

## Declaration of generative AI and AI-assisted technologies in the writing process

During the preparation of this work, the authors used ChatGPT 4o to improve readability, to review the language and to check that the text complies with the recommendations for authors. After using this tool/service, the authors reviewed and edited the content as needed and takes full responsibility for the content of the publication.

## Ethics statement

This study involves human participants and was approved by an Ethics Committee(s) or Institutional Board(s) (IRB Adène, Number IRB_ADENE_20240102). This study does not involve animal subjects.

## Conflict of interest

JUB received travel and congress fees from Gilead, Abbvie, Chiesi and Astellas.

The other authors disclose no conflicts.

Please refer to the accompanying ICMJE disclosure forms for further details.
