# Supplementary material for: Disparities in transplant access and outcomes after first cirrhosis decompensation in alcohol-related liver disease
Source: JHEP Rep. 2025 Sep 18;7(12):101594. doi: 10.1016/j.jhepr.2025.101594 (PMC12639832; doi:10.1016/j.jhepr.2025.101594)
Supplement: Multimedia component 1 [file mmc1.pdf]

Disparities in transplant access and outcomes after first cirrhosis decompensation in alcohol-related liver disease

José Ursic Bedoya, Charlotte De Choudens, Margaux Delhomme, Astrid Herrero, Stéphanie Faure, Lucy Meunier, Magdalena Meszaros, Georges-Philippe Pageaux, Claire Duflos

Table of contents

Supplementary methods: 2

Fig. S1 4

Table S1 5

Table S3 7

Table S4 8

Table S5 9

Supplementary methods:

Frailty model construction:

We excluded the variable attending physician declaration as it had a p-value > 0.10 in univariate models. We did not keep the variables linked to alcohol addiction follow-up as they were too related to the alcohol-related background. We also excluded the variable at least three consultations with a general practitioner in the previous year as it did not meet the proportional hazard assumption.

Using the backward method, we excluded hepatitis B and C and distance from home to nearest transplant centre as they were not statistically associated with transplantation in the multivariate model.

Sensitivity analyses results:

Some important variables (sex and type of centre) did not meet the proportional hazard assumption. We decided to include them still in the model and to perform sensibility analyses to confirm our results. To do so, we built several frailty models at various follow-up time. It allowed us to have multiple hazard ratio at different given times to check for a potential change in the direction of the statistical association.

We kept the same variables and built the model with follow-up up to 6 months, then from 6 months to one year, from 1 to 2 years and beyond 2 years.

As shown in table 4, at every period studied, being a woman reduced the instantaneous risk of being transplanted. The hazard ratio decreases over time, from 0.948 at 6 months to 0.568 after 2 years. This shows that there is no qualitative bias: although women are probably less disadvantaged in terms of access to transplants at the start of follow-up, there is no point at which they are over-transplanted compared with men.

Similarly, having been hospitalized in a transplant centre or a university hospital at the time of first cirrhosis decompensation, rather than in another public hospital, was associated with a higher instantaneous risk of transplantation at all periods studied. We can see that being hospitalized in a transplant centre has a very strong effect before 6 months, with an HR of 3.395, then the HR decreases progressively to reach a level similar to that of other university hospitals after 2 years of follow-up (HR of 1.363 for university hospitals vs. 1.315 for transplant centres). There is no qualitative bias either.

Standardized incidences details:

For standardization, we used the variables gender, MASH, ALD declaration, at least one gastroenterologist consultation in the previous year, type of centre, beneficiary of a complementary health insurance, Charlson index, FDEP15 and age. The Charlson index and FDEP15 variables were divided into quartiles, and age into 5-year increments.

Frailty model with another approach to detect MASH patients:

Secondly, as the K758 code is not always correctly filled in, we built a frailty model by changing the definition of our MASH variable to make it more specific. We therefore considered that a patient had MASH-related cirrhosis when he or she had at least one hospital

stay with K758 as principal, related or associated diagnosis since 2010 and up to one year after their first hospitalization for decompensated cirrhosis and was diabetic according to Pathology and expenditure mapping. The results are shown in Table S4. The model is similar to the one presented in the article. The hazard ratio for MASH is higher than in our first model, but the direction of the association does not change and there is barely any impact on the other multivariate hazard ratios.

Fig. S1: Diagram of SNDS tables method of linking

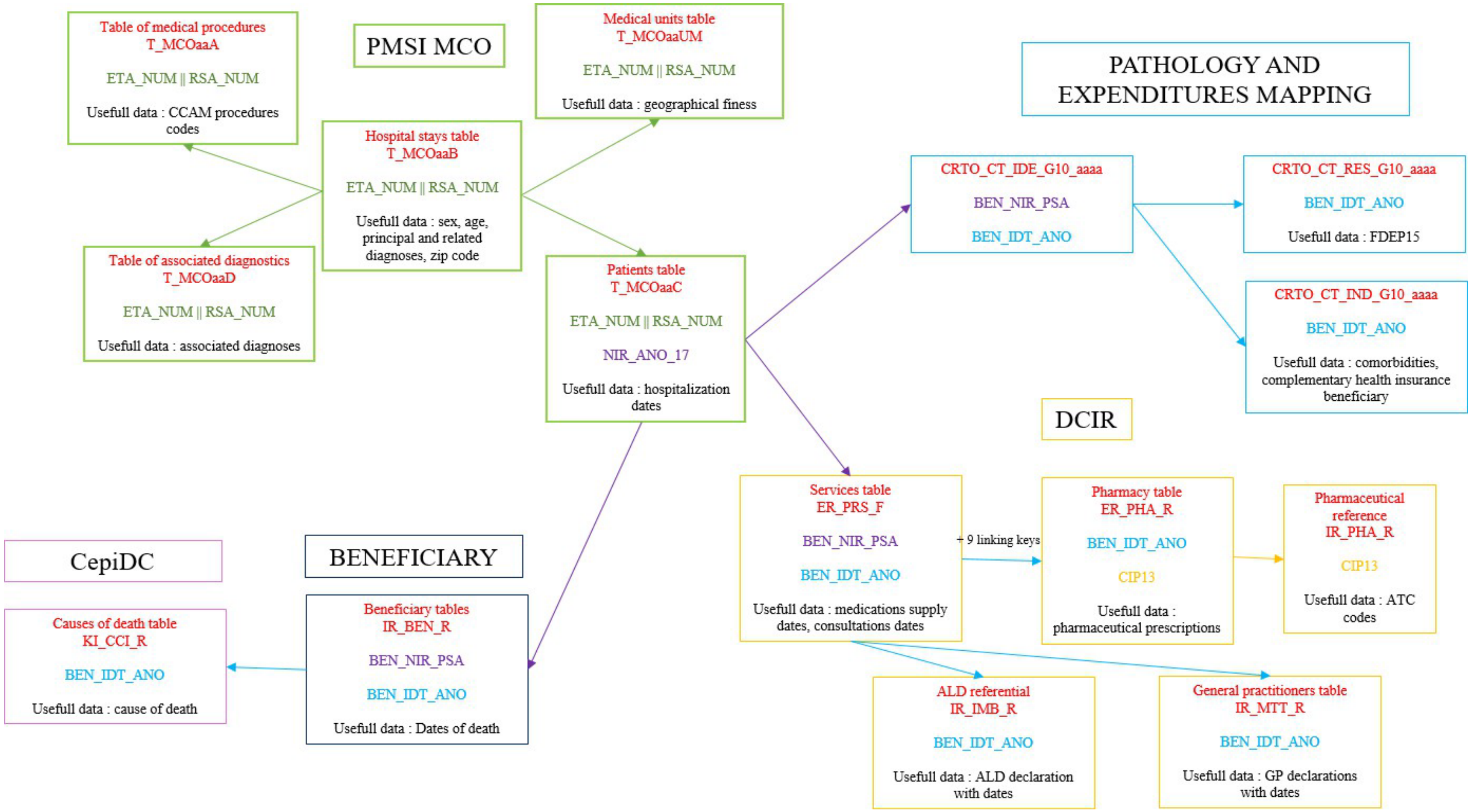

Fig. S1: Diagram of SNDS tables method of linking

Table S1: Charlson index construction:

**Charlson Index**			
Variables / codes used	Source of data	Condition	Weight
	PMSI	Age	+1 for each decade beyond age 50
sup_RIRCT_cat or sup_RIRCT_cat	Pathology and expenditure mapping	Chronic kidney disease	1
sup_CvIC_cat	Pathology and expenditure mapping	Congestive heart failure	2
top_CvAOMI_ind	Pathology and expenditure mapping	Peripheral vascular disease	1
sup_CvAVC_cat	Pathology and expenditure mapping	Cerebrovascular disease	1
top_NDemenc_ind	Pathology and expenditure mapping	Dementia	2
top_ABPCOIr_ind	Pathology and expenditure mapping	Chronic respiratory insufficiency	1
G041, G114, G801, G802, G81, G82, G830-G834, G839	PMSI (ICD-10)	Hemiplegia	2
Excluded by definition		Mild liver disease	2
Considered true by definition		Moderate to severe liver disease	3
sup_CanSur_cat or sup_CanAct_cat	Pathology and expenditure mapping	Cancer (excluding patients with metastatic cancer)	2
C77-C80	PMSI (ICD10)	Metastatic solid tumor	11
top_IRVih_ind	Pathology and expenditure mapping	HIV / AIDS	1

Table S2: details of events defining cirrhosis as decompensated

ICD-10 Code	Diagnosis
A418	Other specified septicemias
A419	Septicemia, unspecified
E807	Mixed acid-base balance disorder
G934	Encephalopathy, unspecified
G92	Toxic encephalopathy
G93	Other cerebral diseases, not elsewhere classified
G94	Other disorders of the brain in diseases classified elsewhere
G96	Other disorders of central nervous system
G98	Other disorders of nervous system, unspecified
G99	Other disorders of nervous system in diseases classified elsewhere
I850	Esophageal varices with bleeding
I983	Hepatorenal syndrome
K650	Acute peritonitis
K658	Other peritonitis
K659	Peritonitis, unspecified
K767	Hepatopulmonary syndrome
K922	Gastrointestinal hemorrhage, unspecified
R170	Hepatomegaly, unspecified
R18	Ascites
R258	Other and unspecified involuntary movements
R400	Somnolence
R401	Stupor
R402	Coma, unspecified
R410	Disorientation, unspecified
R418	Other symptoms and signs involving cognitive functions

Table S3: Summary of frailty models at multiple follow-up times

	HR at 6 months	HR 6 Months to 1 Year	HR 1 to 2 Years	HR > 2 Years	Overall
Number of patients analyzed (number of transplant)	**60,071 (1157)**	**43,189 (623)**	**38,725 (819)**	**33,399 (727)**	**60,071 (3,326)**
**Age**	0.969	0.998	0.998	0.980	0.983
**Sex (reference=men)**	0.948	0.825	0.605	0.568	0.750
**Fdep15**	0.976	0.931	0.956	0.950	0.967
**Beneficiary of solidary Complementary health insurance**	0.788	0.949	0.804	0.783	0.817
**Charlson index**	0.990	1.005	1.000	0.868	0.978
**Alcohol-related background**	0.670	0.801	0.855	0.716	0.748
**MASH**	1.387	1.729	1.187	1.691	1.461
**Declaration of long-term illness**	2.277	2.135	2.054	2.171	2.172
**At least one gastroenterologist consultation in the previous year**	1.673	1.920	1.642	1.579	1.705
**Type of centre (reference = Public hospital)**					
Liver transplant centre	3.395	2.644	1.534	1.315	2.257
University hospital	1.553	1.821	1.237	1.363	1.479
PRIVATE HOSPITAL	0.870	0.912	0.965	0.970	0.927

Table S4:

TABLE S4. FRAILTY MODEL WITH MASH CODED AS ONE STAY WITH K758 + DIABETIC PATIENT ACCORDING TO THE PATHOLOGY AND EXPENDITURE MAPPING

VARIABLE	Hazard Ratio	95% Confidence interval	p-value
**Age**	0.98	0.98 - 0.99	<.0001
**Female sex**	0.75	0.69 - 0.81	<.0001
**FDEP15**	0.97	0.95 - 0.99	0.01
**Beneficiary of public health insurance**	0.82	0.74 - 0.90	<.0001
**Charlson index**	0.98	0.96 - 1.00	0.045
**Alcohol-related cirrhosis**	0.75	0.69 - 0.81	<.0001
**MASH**	1.75	1.32 - 2.33	0.0001
**Declaration of long-term illness**	2.18	2.03 - 2.34	<.0001
**At least one gastroenterologist consultation in the previous year**	1.70	1.58 - 1.83	<.0001
**Type of centre (reference=public hospital)**			<.0001
Liver transplant centre	2.26	1.91 - 2.67	<.0001
University hospital	1.48	1.28 - 1.72	<.0001
Private hospital	0.93	0.82 - 1.06	0.27

Table S5: Cumulative Incidence of Liver-Related Death at 1 and 5 Years by Transplantation Status and Cirrhosis aetiology

	Cumulative incidence function at 1 year [CI95%]	Cumulative incidence function at 5 years [CI95%]
**Non transplanted and alcohol-related cirrhosis**	27.31% [26.90; 27.72]	37.97% [37.51; 38.43]
**Non transplanted and non-alcohol-related cirrhosis**	15.98% [15.26; 16.72]	21.13% [20.30; 21.98]
**Transplanted and alcohol-related cirrhosis**	2.33% [1.79; 2.98]	7.57% [6.53; 8.70]
**Transplanted and non-alcohol-related cirrhosis**	3.33% [2.29; 4.68]	6.65% [5.08; 8.49]
Gray's test for equality of Cumulative Incidence Functions: p<0.0001		
